# Group B Streptococcus Primary Pyogenic Ventriculitis in an Adult

**DOI:** 10.7759/cureus.63954

**Published:** 2024-07-06

**Authors:** Sangam Sangam, Michael Xerras, Shivam Khatri, Shorabh Sharma, Michelle Dahdouh

**Affiliations:** 1 Internal Medicine, St. Barnabas Hospital Health System, New York, USA; 2 Internal Medicine, City University of New York (CUNY) School of Medicine, New York, USA; 3 Medicine, City University of New York (CUNY) School of Medicine, New York, USA; 4 Infectious Disease, St. Barnabas Hospital Health System, New York, USA

**Keywords:** infectious disease pathology, gbs, group b streptococcus (gbs) primary pyogenic ventriculitis, s. agalactiae, pyogenic ventriculitis

## Abstract

Pyogenic ventriculitis is a disorder characterized by inflammation of the cerebral ventricular lining secondary to infection within the ventricular system. Very few cases of primary pyogenic ventriculitis have been reported among adults. We present a case report of a 74-year-old female with a history of hypertension and diabetes mellitus who presented with Group B Streptococcus (GBS) primary pyogenic ventriculitis. She was successfully treated with intravenous (IV) antibiotics. To our knowledge, this is the only case of adult *Streptococcus agalactiae* primary pyogenic ventriculitis.

## Introduction

Pyogenic ventriculitis is a suppurative infection of the cerebral ventricular lining [1]. Cases occur most commonly in children and infants due to the extension of infection from bacterial meningitis or otitis media. Cases of pyogenic ventriculitis are uncommon in adults and can occur secondary to trauma, ventricular drain, shunt, surgery, ruptured abscess, immunocompromised state, or meningitis. Organisms that commonly cause ventriculitis include staphylococcus species, meningococcus, and gram-negative bacillus. Pyogenic ventriculitis usually manifests with symptoms such as fever, altered mental status, headache, nausea, and photophobia/phonophobia. Meningeal signs are common, including Kernig/Brudzinski signs and nuchal rigidity [2-4].

Only a few cases of primary ventriculitis have been reported, most of them being Group B streptococci neonatal infections. Only six of these cases have been described in adults, including only one due to *Neisseria meningitidis* [5]. The median age of patients with pyogenic ventriculitis is 65 years [5]. Among reported cases of ventriculitis, gram-negative organisms were the cause in 60% of cases. The next most common cause was staphylococcal species [6]. Cases due to *Streptococcus pneumoniae*, *Hemophilus influenzae*, and *Streptococcus pyogenes* have mostly been reported in patients secondary to skull base fractures and persistent cerebrospinal fluid (CSF) leaks [2]. To our knowledge, no cases of adult primary pyogenic ventriculitis due to Group B Streptococcus (GBS) without associated endocarditis exist.

## Case presentation

A 74-year-old female with a past medical history of hypertension, diabetes mellitus, hyperlipidemia, osteopenia, constipation, hypothyroidism, asthma, Sjogren’s syndrome, monoclonal gammopathy of unknown significance with cryoglobulins, and *Helicobacter pylori* infection presented to the emergency department (ED) with a two-day history of intermittent tactile fevers. The patient also reported an intense headache associated with photophobia, chills, abdominal pain, and non-bilious, non-bloody vomiting, which had been going on for three days.

Upon initial examination, her vital signs were recorded as follows: a temperature of 98.8 °F (37.1 °C), a heart rate of 97 beats per minute, a respiratory rate of 18 breaths per minute, and a blood pressure of 188/88 mmHg. She appeared drowsy but responded appropriately to verbal cues by opening her eyes and demonstrating proper orientation to person, place, and time. Kernig's and Brudzinski's signs were both negative; however, passive flexion of the neck elicited pain.

**Figure 1:**
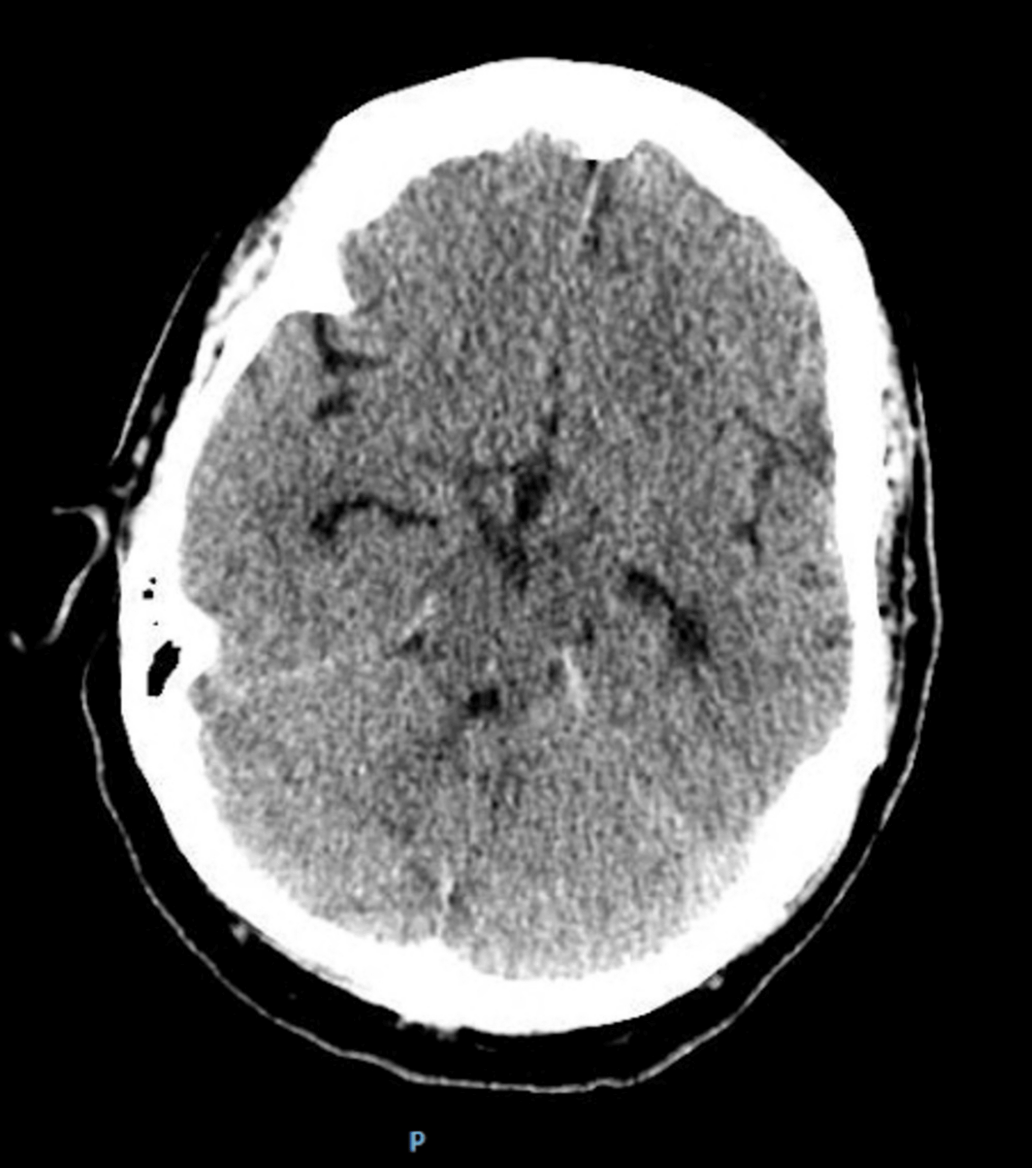
Non-contrast computed tomography (CT) scan of the head showed no acute intracranial abnormality.

Initial laboratory tests revealed leukocytosis with lymphopenia, neutrophilia, and eosinophilia, along with hypokalemia (Tables 1, 2). A non-contrast computed tomography (CT) scan of the head was performed, which ruled out any acute intracranial abnormality (Figure 1). She was administered intravenous (IV) ampicillin, ceftriaxone, vancomycin, and dexamethasone in the ED. Additionally, potassium was provided to correct the hypokalemia.

**Table 1:** Complete blood count throughout the duration of the hospital stay. ED: emergency department; WBC: white blood cell; RBC: red blood cell

Test	Normal Range	ED	Day 9	Day 11
WBC (10^3^/uL)	4-10	28.6	11.1	6.8
Platelets (10^3^/uL)	150-450	299	400	363
Hemoglobin (g/dL)	11.2-15.7	12.7	13.3	11.9
RBC (10^6^/uL)	3.93-5.22	4.43	4.60	4.09

**Table 2:** Basic metabolic panel throughout the duration of stay. ED: emergency department

Variables	Normal Range	ED	Day 9	Day 11
Sodium (mEq/L)	135-145	133	135	135
Potassium (mEq/L)	3.5-5.3	2.8	4.2	4.0
Chloride (mEq/L)	96-108	98	99	103
Glucose (mg/dL)	70-99	204	108	115
Urea nitrogen (mg/dL)	8-23	20	12	7
Creatinine (mg/dL)	0.6-1.2	0.8	0.7	0.7
Calcium (mg/dL)	9.2-11.0	9.0	8.4	8.6

**Figure 2:**
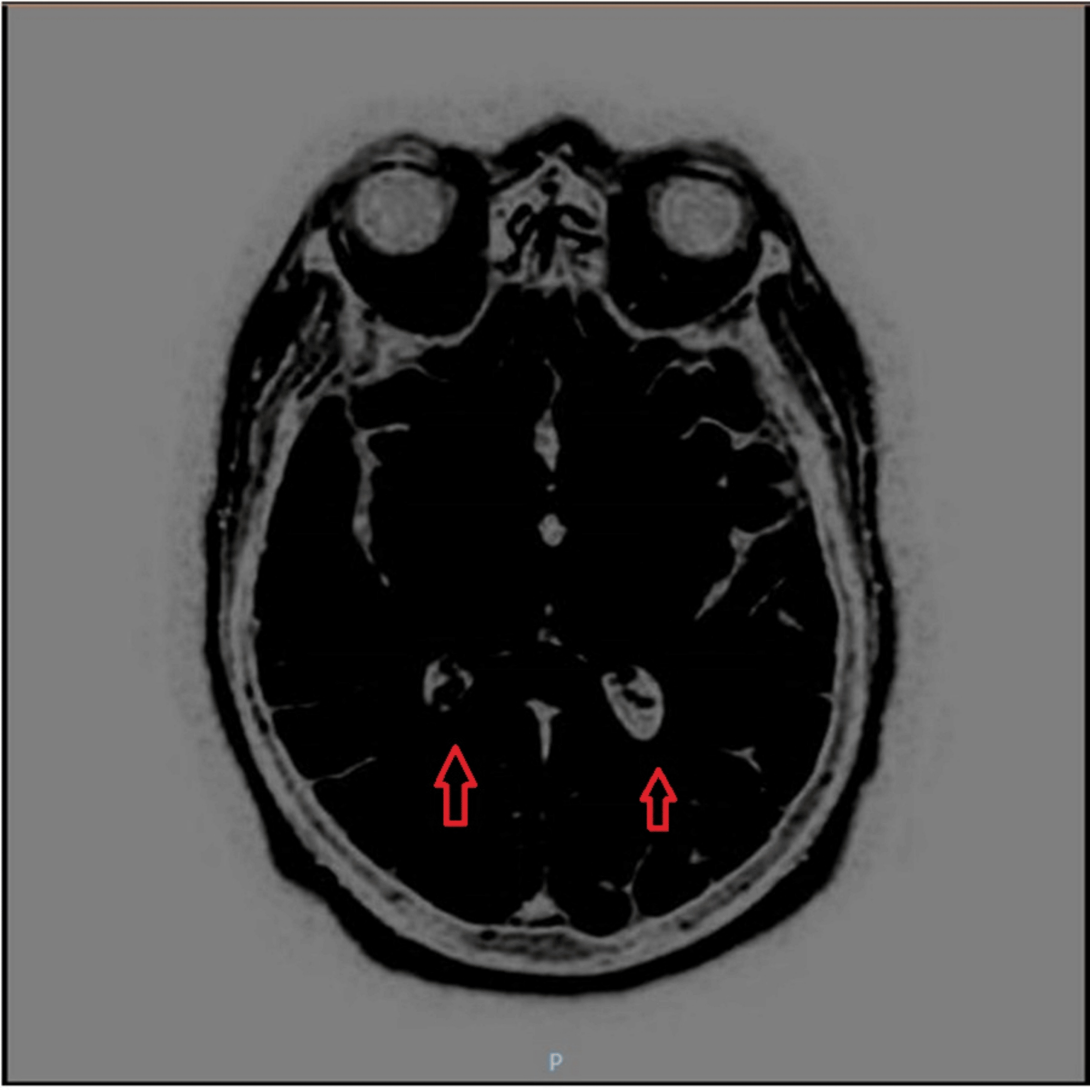
A contrast-enhanced MRI of the brain was conducted, revealing fluid levels with increased diffusion and FLAIR signal within the occipital horns of bilateral lateral ventricles, which was suggestive of pyogenic ventriculitis. FLAIR: fluid-attenuated inversion recovery; MRI: magnetic resonance imaging

The patient was admitted with a preliminary diagnosis of meningoencephalitis. An initial attempt to perform a lumbar puncture did not yield any CSF. Consequently, the patient was started on empiric antibiotic therapy, receiving ampicillin, ceftriaxone, vancomycin, and acyclovir. Dexamethasone was also administered but was later discontinued after four days of treatment. Subsequently, a fluoroscopy-guided lumbar puncture was performed, and the CSF analysis raised suspicion for pyogenic meningitis on day two of hospitalization (refer to Table 3 for specific findings). To further investigate the condition, a contrast-enhanced magnetic resonance imaging (MRI) of the brain was conducted, revealing fluid levels with increased diffusion and fluid-attenuated inversion recovery (FLAIR) signal within the occipital horns of bilateral lateral ventricles, which was suggestive of pyogenic ventriculitis (Figure 2).

**Table 3:** Cerebrospinal fluid analysis. WBC: white blood cell; RBC: red blood cell

Characteristic/Value	Normal Range	Result
Color	Colorless	Yellow
Appearance	Clear	Cloudy
Xanthochromia	Negative	Positive
WBC (/mm^3^)	0-5	7220
RBC (/mm^3^)	0-0	805
Neutrophil (%)	0-6	92
Lymphocyte (%)	40-80	3
Monocyte (%)	15-45	5
Protein (mg/dL)	40-70	59
Glucose (mg/dL)	15-45	371

**Figure 3:**
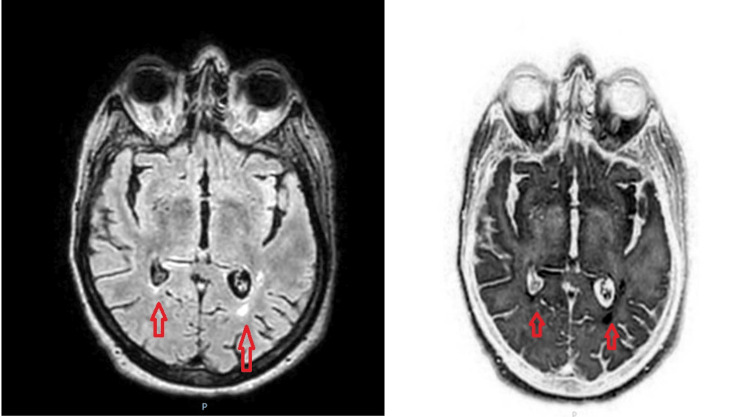
Repeat magnetic resonance imaging (MRI) of the brain and T2 sagittal and coronal sequences indicated persistent pyogenic ventriculitis.

Over the course of the following days, the patient displayed signs of improvement and started tolerating oral feeding. Meanwhile, CSF studies revealed a positive polymerase chain reaction test for GBS, confirming the diagnosis and prompting a narrowed antibiotic coverage of ceftriaxone and metronidazole for a total duration of six weeks; the latter was included to address any possible brain abscesses. Blood cultures showed no growth. Additional imaging studies, including CT without contrast of the brain with thin slices and CT maxillofacial and CT internal auditory canal (IAC) to visualize the petrous bone, were conducted to evaluate for possible sources of infection, including the sinuses or a ruptured brain abscess. CT maxillofacial was remarkable for mild mucosal disease of the left sinus, and CT IAC showed mild discontinuity of the cribriform plate medially with slight herniation of CSF, possibly a frontoethmoidal meningocele. Subsequent repeat MRI of the brain and T2 sagittal and coronal sequences indicated persistent pyogenic ventriculitis (Figure 3) with an anterior skull base bony defect and a very small encephalocele in the midline. To further assess for CSF leak, an outpatient radionuclide cisternogram was recommended, along with monitoring of erythrocyte sedimentation rate (ESR) and C-reactive protein (CRP) and serial MRIs.

Throughout the treatment period, the patient showed remarkable improvement. Her fever resolved, and the white blood cell (WBC) counts started to decrease, eventually returning to normal on the 11th day of her hospital stay. Additionally, the CRP levels decreased significantly from 26.16 to 1.65, indicating a positive response to the antibiotic therapy. A transthoracic echocardiogram (TTE) was done, which did not show any signs of endocarditis. A transesophageal echocardiogram was offered, but the patient's family refused. As her condition stabilized, the patient was discharged to a skilled nursing facility with a midline in situ, where she completed the remaining six weeks of intravenous antibiotic treatment. Subsequently, the patient attended regular follow-up evaluations at an Infectious Disease clinic. During the follow-up period, a repeat brain MRI confirmed the complete resolution of pyogenic ventriculitis, signifying the successful treatment outcome. The patient eventually decided to retire to Florida and is now under the care of her primary care provider for ongoing medical management and monitoring.

## Discussion

Pyogenic ventriculitis is an uncommon manifestation of a severe intracranial infection that is rare in adults. The condition can be caused by various factors, including trauma, surgery, or the presence of a foreign object such as a catheter [4,7]. However, primary ventriculitis is very rare, especially in adults [8]. Primary ventriculitis is typically caused by organisms such asmeningococcus and staphylococcus. A recent literature review revealed no reported cases of Group B streptococcal ventriculitis [5]. The case being discussed presents a scenario that is the first of its kind, as it involves a 74-year-old female who presents with Group B streptococcal primary pyogenic ventriculitis without associated endocarditis.

The exact pathogenesis of ventriculitis is not fully understood, but it is thought to involve bacterial colonization and invasion of the ventricular system. If not diagnosed and treated in time, it can be life-threatening and neurologically disabling.

The neuroradiological findings of pyogenic ventriculitis may vary. An MRI of the brain plays a major role in diagnosing ventriculitis. MRI features specific to ventriculitis include ventricular debris, hydrocephalus, ventricular hyperintense signals, and ependymal enhancement [1,6]. According to a study by Fukui et al., ventricular debris was detected in 94% of cases. The debris was hyperintense to CSF on T1-weighted images and hypointense to CSF on T2-weighted images. Hydrocephalus was detected in 76% of cases. Periventricular hyperintensities were present in 78% of cases with MRI and were most conspicuous on FLAIR images. Ependymal enhancement was detected in 64% of cases where contrast material was administered [9]. MRI can show pus in the dependent position of the occipital horns with a marked hyperintense signal compared with CSF and the brain [10]. In a case reported by Jayndrakumar et al., the patient’s brain MRI exhibited intraventricular T2/FLAIR hyperintensity with mild diffusion restriction and fluid‑fluid level in the dependent portion of the occipital horn of the bilateral ventricle (Figure 2). In our case, the patient showed similarities as the MRI with contrast exhibited fluid levels with increased diffusion and FLAIR signal within the occipital horns of the bilateral lateral ventricles. However, the patient in this case deviated from the typical radiological presentation as a small anterior skull base bony defect with a very small encephalocele in the midline was found on MRI without contrast [6].

There is a case report in the literature involving an adult presenting with GBS pyogenic ventriculitis secondary to endocarditis. Adbelradi et al. reported a case of a 69-year-old female with a history of type 2 diabetes mellitus who presented with a progressively worsening course before admission. On physical examination, she was lethargic but arousable and had no nuchal rigidity. She was treated for diabetic ketoacidosis (DKA) with intravenous insulin and fluid resuscitation. She was also started on empiric ceftriaxone and vancomycin due to severe sepsis. On the second day of hospitalization, the patient became increasingly lethargic and was minimally responsive. Blood cultures from admission grew GBS. A lumbar puncture was performed, and analysis of the CSF showed a WBC count of 4,500/mm^3^, a protein of 354 mg/dL, and a glucose level of 140 mg/dL. Antibiotics were switched to penicillin plus gentamicin. A transthoracic echocardiogram revealed 0.5 cm vegetation on the anterior leaflet of the mitral valve with mild mitral regurgitation. The patient underwent an MRI of the brain, which showed scattered foci of FLAIR hyperintensity within the bilateral cerebral sulci and ependyma, suggestive of meningitis and ventriculitis. The patient received an initial two weeks of penicillin G plus gentamicin, followed by an additional two weeks of penicillin G alone. Repeat blood cultures showed no further growth of the GBS [2].

There is another case report in the literature involving an adult presenting with sudden hearing loss who was diagnosed with pyogenic ventriculitis caused by GBS* *endocarditis. Toshiki et al. reported a case of a 54-year-old Asian female who presented with sudden hearing loss in her left ear, which had developed over the course of two days. She had no history of ear disease or trauma, and no other symptoms were reported. Physical examination revealed no abnormalities except for a mild fever (37.5 °C). Pure-tone audiometry showed severe sensorineural hearing loss in the left ear. MRI of the brain revealed a small abscess in the left lateral ventricle, which was confirmed by a CT scan. Blood cultures were positive for *S. agalactiae*, and TTE showed vegetation on the mitral valve, indicating endocarditis. The patient was treated with intravenous antibiotics, including vancomycin and ceftriaxone. She underwent mitral valve replacement surgery and ventricular drainage, which successfully resolved the abscess. The patient fully recovered, except for hearing loss, approximately six months after surgery [3].

The treatment of pyogenic ventriculitis requires prompt and aggressive therapy with antibiotics and surgical intervention when necessary. The choice of antibiotics should be based on the culture and sensitivity testing results. In some cases, intraventricular administration of antibiotics may be necessary [9]. In the case of Adbelradi et al., the patient received an initial two weeks of penicillin G plus gentamicin, followed by an additional two weeks of penicillin G alone [2]. In the case of Toshiki et al., the patient received IV vancomycin and ceftriaxone, as well as ventricular drainage, which successfully resolved the abscess [3]. These differ from the patient in our case, who received IV ceftriaxone and IV metronidazole for six weeks. However, the cases are similar as both patients improved significantly while receiving treatment, and MRI showed resolution of the ventriculitis in both cases.

## Conclusions

Adult primary pyogenic ventriculitis secondary to GBS has not yet been documented in the literature. Only neonatal GBS pyogenic ventriculitis and adult GBS pyogenic ventriculitis secondary to GBS endocarditis have been documented in the literature. Gaining a comprehensive understanding of the neuroradiological findings and potential treatment approaches for this condition can significantly benefit infectious disease specialists and neuroradiologists in promptly identifying the diagnosis, leading to improved patient outcomes.
